# On “Manual Therapy in Preadolescent Children: A Delphi Investigation of Physical Therapists in the United States.” Dice JL, Dendy D, Sizer PS, Cook CE, Feuling S, Brismée JM. *Phys Ther*. 2021;101:pzab027. https://doi.org/10.1093/ptj/pzab027

**DOI:** 10.1093/ptj/pzac114

**Published:** 2022-08-11

**Authors:** Gail Wetzler, Thomas R Rasmussen, Leonard A Wisneski, Dawn L Shear

**Affiliations:** Department of Research, Upledger Institute International and Barral Institute, Palm Beach Gardens, Florida, USA; Department of Research, Upledger Institute International, Palm Beach Gardens, Florida, USA; Center for Manuel Medicine, Vaerlose, Denmark; Department of Medicine, University of Colorado, Denver, Colorado, USA; International Alliance of Healthcare Educators, Research and Development, Palm Beach Gardens, Florida, USA

**Keywords:** CranioSacral Therapy, Manual Therapy, Pediatrics, Physical Therapy Modalities, Physical Therapy Specialty, Visceral Manipulation

This letter comments on an original research article titled, “Manual Therapy in Preadolescent Children: A Delphi Investigation of Physical Therapists in the United States,” by Dice et al.^1^ The objective stated in the article was to gather expert opinion, frame future studies, and assist in guiding clinical practice toward the advantages of manual therapy used by physical therapists for preadolescent children. Our comments concern the consensus and stated result that visceral manipulation and craniosacral therapy were considered ineffective in treating most impairments:

(1) The inclusion of craniosacral therapy (CST) and visceral manipulation (VM) as part of the list of manual therapy techniques noted as “other” techniques was apparently done to allow respondents to add techniques; it was not included in the initial list.

(2) These techniques were added by very few randomly selected therapists. We indicate “few,” because in the discussion the authors stated that several respondents from each group answered, “I am not familiar with this technique.” The article gives no data on this fact; we, therefore, speculate that only a few of the respondents might have possessed adequate knowledge regarding these techniques.

(3) The authors were quite clear that this group maintained advanced credentials in pediatrics, neurodevelopment, and manual therapy. None of the groups mentioned board certification as a clinical specialist in pediatric physical therapy, certification in NDT (C/NDT), nor credentials in manual therapy (Fellow of the American Academy of Orthopaedic Manual Physical Therapists [FAAOMPT]) nor included continuing education or certification in CST or VM.

(4) There is no data expressing the clinical expertise of the randomly chosen therapists having experience with CST or VM. Proof of applied knowledge and skill level is paramount to being able to render an accurate assessment of a discipline.

(5) There is no mention of CST or VM in the graph of selected manual therapy approaches.

(6) We question whether the authors are, therefore, in a position to state conditions or conclusions for which there is no data. In scientific research, the methods used should be adequate for the research questions being asked, and conclusions should be drawn clearly from the data presented in the study. If the authors could show data supporting the conclusions regarding CST and VM in the paper, it could clarify the obvious misleading conclusions in the paper (see below). If the authors cannot present conclusive data on the ineffectiveness of CST and VM, a withdrawal of the conclusion in a response letter would promote the development of science in manual therapy.

(7) The discussion section stated: “There was strong consensus that craniosacral therapy and visceral manipulations were not perceived as effective in treating decreased proprioceptive awareness, hypotonicity, joint pain, and spasticity by those who used the Likert scale.”

Did any of the respondents evaluate this loss of proprioceptive awareness, hypotonicity, joint pain, or spasticity as having a root cause to dural, neuromeningeal, or organ suspensory systems? If not, they did not possess knowledge of CST or VM, and these techniques should not have been considered in this study.^2–5^

We are grateful that the authors stated, “In the clinic, physical therapists use clinical experience combined with critical thinking and sound clinical reasoning to implement manual therapy techniques.” It appears to us that this is not the case with this research, however. It is apparent that there was an absence of clinical experience or critical thought process, which led to the conclusion that CST or VM was ineffective in treating most impairments in preadolescent children.^6^
